# Phenotypic Features in a New 12q21 Deletion and Its Association With Cardiofaciocutaneous Syndrome

**DOI:** 10.7759/cureus.59831

**Published:** 2024-05-07

**Authors:** Indushree Manjunath, Arturo Gamez, Anitha Jagadish

**Affiliations:** 1 Medical Intern, Sapthagiri Institute of Medical Sciences and Research Center, Bengaluru, IND; 2 Pediatric, Tender Care Pediatrics, Port St. Lucie, USA; 3 Pediatric Medicine, Tender Care Pediatrics, Port St. Lucie, USA

**Keywords:** 12q21 syndrome, pediatric, genetic, cardiofaciocutaneous syndrome, 12q21 deletion

## Abstract

Arriving at a diagnosis in children with developmental delay, cognitive impairments, and multiple physical abnormalities at birth can be very taxing due to many differential diagnoses and etiologies. Of the plethora of conditions that are seen among infants, chromosomal disorders, in particular, present with challenges in diagnosis and devastating consequences. In recent times, the advent of chromosomal microarray techniques has made it possible to easily identify chromosomal deletions and arrive at a diagnosis. This case comprises one of the very few cases reported in interstitial deletions of the long arm of chromosome 12. To date, only 14 patients with deletions, including the 12q21 region, have been reported. The main features are cardiac, renal, ocular, CNS, and developmental abnormalities. The shared features of all these cases might suggest a possible microdeletion syndrome. In this case report, we propose through descriptive analysis that a deletion of genes in the 12q21 region could lead to CFC syndrome. This work contributes to our understanding of the 12q21 deletion syndrome through the case discussion of a one-year-seven-month-old boy with a de novo deletion at 12q21.1q21.31 region that has never been reported previously.

## Introduction

Interstitial deletion on the long arm of chromosome 12 in the 12q21 region has been described in around 14 patients so far. Although the patients with 12q21 deletion share some similarities, such as developmental delay, cognitive impairments, and multiple physical abnormalities at birth, they have different phenotypic presentations. The differences in phenotypes are mostly a consequence of the size and the locus of the deletion. As the deletions tend to be larger, it’s seldom possible to determine which gene is the exact cause of the individual phenotypic manifestations. However, this report talks about some important genes that were found to be closely associated with the phenotypic manifestations in our patient as well as other patients with similar clinical manifestations. 

In this study, we describe a one-year-seven-month-old boy with cardiac defects, renal anomalies, and developmental delay with no known family history of genetic abnormalities. The genotypes and phenotypes of patients from previous reports have been compared with our patient to provide a better understanding of the minute differences in the manifestations of the cardiofaciocutaneous (CFC) syndrome while also throwing light on some stark similarities between them, thus serving to support the theory that deletions in the 12q21 region could be a part of a larger syndrome, which has been accounted in previous reports [1]. 

It is of great interest that the deleted region is positioned in the 12q21.1q21.31 region of chromosome 12, which, to our knowledge, has not been reported and described previously. 

We hope to facilitate a better understanding of this syndrome through our case report and aid clinicians in arriving at a clinical diagnosis based on phenotypic findings.

## Case presentation

In this case report, we describe a one-year seven-month-old boy who is the only child of non-consanguineous parents. The mother had chronic hypertension treated with labetalol 400 mg thrice daily throughout her pregnancy. There was no history of radiation or teratogenic drug exposure during her pregnancy. Concerns about fetal development began when antenatal ultrasound scans revealed that the fetus had bilateral hydronephrosis with no calyceal dilation or parenchymal changes. Due to fetal risk because of maternal hypertension, the mother was planned for elective lower segment cesarean section, for which she received one dose of steroids for fetal lung maturity. Two weeks later, the fetal evaluation revealed a biophysical profile of six/eight (two points deducted on account of breathing difficulty) and a non-reactive non-stress test. She underwent a lower segment cesarean section on the same day, and the baby was born at 31 weeks two days and weighed 1409g. 

His appearance, pulse, grimace, activity, and respiration score (APGAR score) after one minute was eight, and after five minutes, it was also eight. Due to respiratory distress, he required Neopuff continuous positive airway pressure right after delivery, and he was admitted to the neonatal intensive care unit immediately. He was on continuous positive airway pressure and received one dose of surfactant to facilitate lung maturity on day one of life. As the respiratory distress didn’t resolve, he had to be escalated to nasal intermittent positive pressure ventilation and eventually required intubation for three days, after which his respiratory distress resolved and was successfully weaned to room air. 

Due to concerning features in the antenatal scans and the possibility of a genetic disorder in the baby, further evaluation was done, which revealed the following findings. An echocardiogram done in the neonatal period showed a mildly dilated right ventricle with good function and a small patent foramen ovale (PFO). The abdominal ultrasound done at the same time incidentally revealed inferior vena cava with reversal of flow. Renal ultrasound showed bilateral severe upper pole hydronephrosis secondary to upper pole ectopic ureters, for which he was advised amoxicillin 20 mg per day for vesicle ureteral reflux prophylaxis. A scrotal ultrasound revealed the right testes in the canal; however, the left testes could not be visualized in the scrotal sac, groin, or abdomen. The various physical features led to the suspicion that there could be an underlying genetic abnormality. Hence, a deoxyribonucleic acid (DNA) microarray was performed on a sample of blood extracted from the peripheries, which revealed a deletion in the 12q21.1q21.31 region of chromosome 12. 

The boy was closely monitored for the next couple of months. His height and weight progressively increased; however, he has always been below the third percentile. Concerns for developmental delay started when he was nine months old and could not support himself while sitting. He was referred to occupational therapy when he was unable to babble, wave, or walk with support at the age of 12 months. He had several delays in gross motor, fine motor, and coordination. He also demonstrated visual motor as well as visual-perceptual delays that hindered his engagement in age-appropriate play.

Follow-up renal ultrasounds continued to show bilateral severe upper pole hydronephrosis secondary to upper pole ectopic ureters, as shown in Video 1. However, a genitourinary examination showed a symmetric scrotum with testis descended bilaterally. Small bilateral hydroceles were present. Follow-up evaluations by the cardiologist at 10 months of age showed spontaneously resolved PFO, which was later diagnosed as a normal variant as it did not contribute to any significant comorbidities in the baby.

**Video 1:** Renal ultrasound showing bilateral severe upper pole hydronephrosis secondary to ectopic ureters We thank our patient and the family for giving us permission to use the following content for our report.

**Figure 1:**
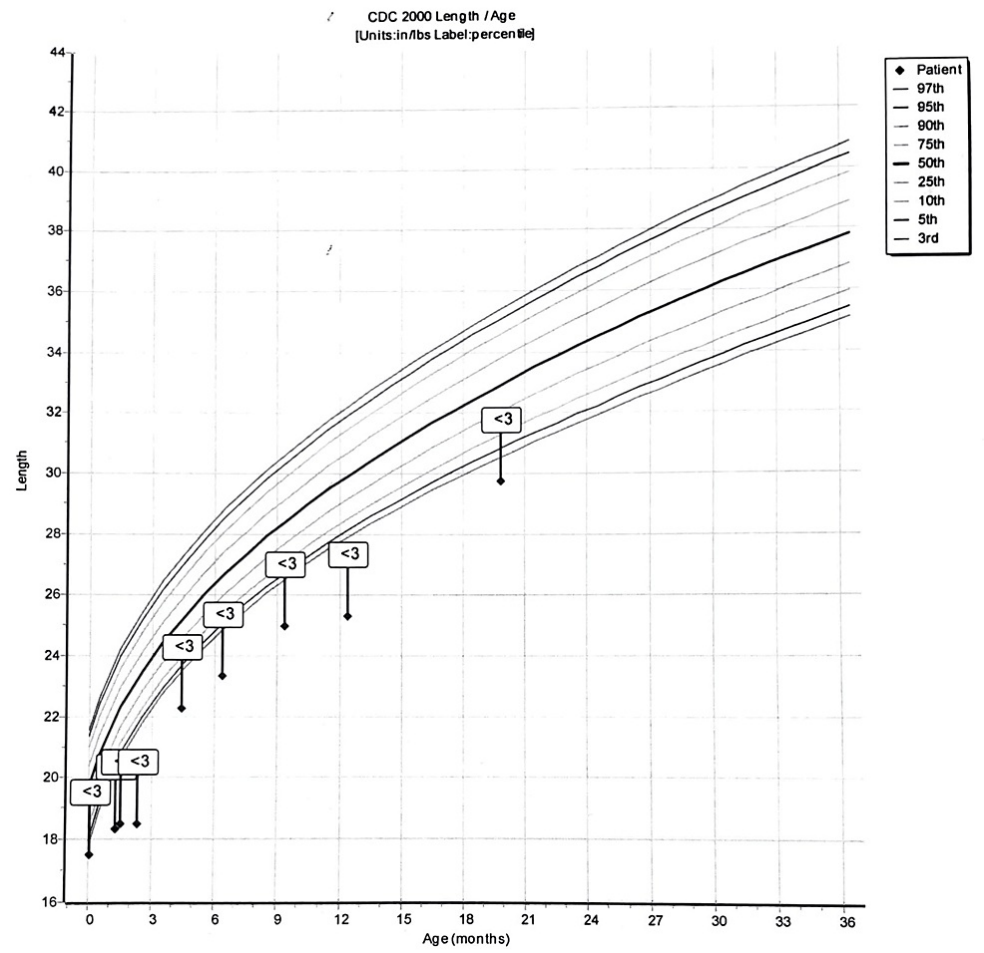
Graph showing the length for age of the patient, which is always tracked along the third percentile

**Figure 2:**
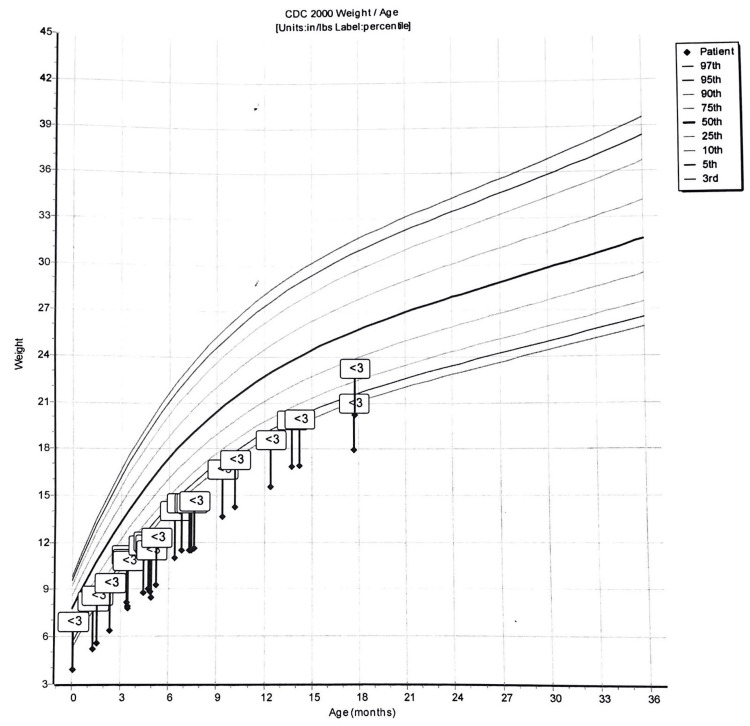
Graph showing the weight for age of our patient, which is always tracked along the third percentile

The child is now in the third percentile for height and weight, as shown in Figures 1, 2. His physical appearance resembled the case reported by Rauen [2], including a prominent forehead, hypertelorism, triangular face, low-set ears, a short nose, developmental delay, bilateral hydrocele, sparse hair, and patent ductus arteriosus.

His genotype and phenotype have been found to be similar to previously reported cases with deletions in the 12q region. The similarities may suggest a syndromic manifestation in patients with a deletion in the 12q region, with minor differences in phenotypes depending on the exact locus of the deletion.

## Discussion

The first case of 12q deletion was reported by Meinecke in 1985, where he reported a child with hypertelorism, broad nasal bridge, and cleft lip and palate and said that the phenotype of the child closely resembled trisomy 18 [3]. However, the understanding of this genetic mutation and its phenotypic manifestation in patients at that point in time was poor. Since then, the advancement of technology in the realm of genetics has facilitated the detection of specific mutations in the 12q21 region and has improved our understanding that there is truly a correlation between such a mutation and the resulting phenotypic manifestation. According to the reviewed literature, all the mutations in this region have some common clinical features, such as development delay, heart defects, and central nervous system anomalies. 

There have been reports from the past where a 15-year-old girl was evaluated for Noonan syndrome (the most common phenotypic features being wide-set eyes, low-set ears, short stature, and pulmonic stenosis) and was later found to have an interstitial deletion in the 12q region [4]. Table 1 lists the distinguishing characteristics between the two syndromes, which have very similar and overlapping phenotypes [5].

**Table 1:** Difference between Noonan syndrome and CFC syndrome CFC, cardiofaciocutaneous

	Noonan syndrome	CFC syndrome
Facial features	Broad forehead, drooping eyelids, wide-set eyes, depressed nose, big forehead, low hairline, and thin transparent skin	The facial features are similar to that of Noonan syndrome but are coarser with the absence of the characteristic blue-green eyes of Noonan syndrome
Skin disorders	Usually, uncommon	Almost always present
Lung disorders	Pulmonic stenosis is seen in most patients	Usually, uncommon
Neurological abnormalities	Very mild	Can progress to severe mental retardation

It was only in the early 2000s that we started to suspect that this phenotypic manifestation could be part of a syndrome called the cardiofaciocutaneous (CFC) syndrome [1]. The use of genomic hybridization (comparative genomic hybridization array) further increased our understanding of the specific locus of the deletion and the possibility that all such deletions could be part of a syndrome. 

The boy whom we have described in this report has features of developmental delay and cardiac abnormalities at birth, which was later diagnosed as mild PFO and dysmorphic facial features (such as frontal bossing, flattened occiput, small sunken eyes, beaked nose, low-set ears, thin upper lip, high arched palate, supraorbital ridges, blepharophimosis, sparse hair, and micrognathia/retrognathia). Similar features were observed and reported previously by Rauen et al. in 2002, who reported a child with developmental delay, patent ductus arteriosus, PFO, and generalized follicular hyperkeratotic papular eruptions [1]. Klein et al., in 2005, also reported similar features of developmental delay, patent ductus arteriosus, PFO, atopic dermatitis, and bi-temporal alopecia [6]. James et al., in 2005, also reported a child with features such as developmental delay, molecular, hyper keratotic, popular eruptions, and dysmorphic facial features [7]. There are more recent reports by Niclass et al. in 2020, where they report a case of developmental delay, heart defects, dermal abnormalities, and dysmorphic facial features [8]. One common feature in all these cases was a mutation in the 12q21 region. All these reports show a strong correlation between the observed features and the possibility of a syndrome manifestation called the CFC syndrome.

Further research led us to understand that genes on the RAS-MAPK pathway are responsible for CFC syndrome. Although associated with the disease pathogenesis, no genes related to this signaling pathway were located within the deleted regions of 12q21. However, the LIN7A, as described in the case report by Matsumoto [9], was located within the deleted region of the 12q21 gene and was found to be associated with intellectual disability. While the gene was not directly involved in neuronal proliferation, the loss of function induced defective migration and axonal growth of excitatory pyramidal neurons during corticogenesis.

Previous reports published in the year 2020 by Niclass et al. described two genes in this region that, when deleted, caused a syndrome similar to our patient. The genes described in this report were SYT1 and PPPR12A [8]. SYT1 is a synaptotagmin protein that plays an important role in mediating the release of calcium-triggered neurotransmitters that are involved in vesicle exocytosis, the dysfunction of which can lead to severe neurological impairment and developmental delay [8,10]. Its deletion can cause a rare deletion called Baker-Gordon syndrome. The PPP1R12A gene encodes a regulatory subunit of myosin phosphatase and was recently implicated in a study conducted in 2019 to be associated with congenital malformations involving the embryogenesis of the brain, the genitourinary system, and the reproductive system [3].

Although 12q21 mutations cause the CFC syndrome, it’s important to note that it’s not the only mutation that can cause this syndrome. Reports show that most patients with the syndrome have mutations in the BRAF, MAP2K1, MAP2K2, or KRAS genes [11]. The purpose of this report is to highlight the fact that there are potentially significant genes located in the 12q21 locus that cause the CFC syndrome as well. However, more evidence is necessary to confirm the causal effect of the gene deletion. It is crucial to further sequence the deleted genes in the 12q21 region to know the exact cause of the clinically observed phenotypes. This will help us have a clear understanding of the syndrome and draw specific correlations between the deleted genes and the syndrome itself. 

**Figure 3:**
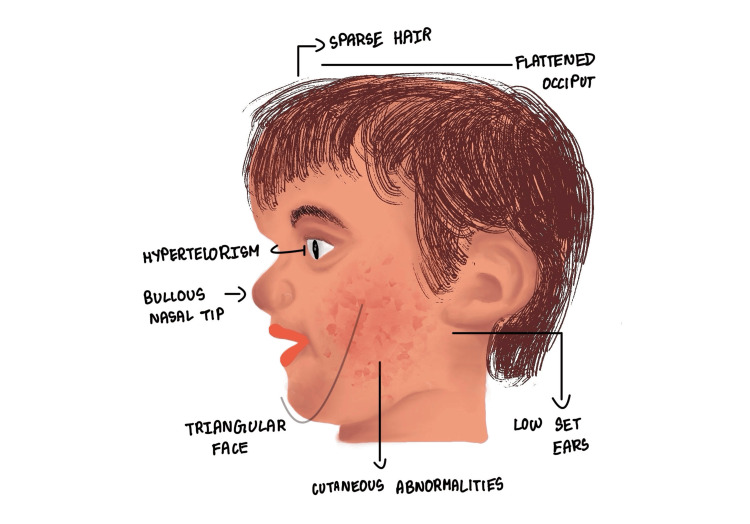
Phenotypic features in a child with 12q21 deletion We thank the patient and his family for allowing us to take inspiration from him to draw this image.

Based on the literature reviewed so far, we propose diagnostic criteria that can possibly be used to diagnose CFC syndrome, particularly among children with 12q21 deletions. Figure 3 helps illustrate the classical phenotypic presentation in children with 12q21 deletion or CFC syndrome. The most commonly observed features are a prominent forehead and flattened occiput, sparse hair, retrognathia, bulbous nasal tip, triangular face, low-set ears, hypertelorism, and cutaneous abnormalities (e.g., eczema). The presence of five or more of the features, as listed in Table 2, can suggest a strong correlation with CFC syndrome.

**Table 2:** The most commonly observed phenotypic features in cardiofaciocutaneous syndrome

Sl. no	Phenotypic features suggestive of cardiofaciocutaneous syndrome
1	Prominent forehead and flattened occiput
2	Sparse hair
3	Retrognathia
4	Bulbous nasal tip
5	Triangular face
6	Low-set ears
7	Hypertelorism
8	Cutaneous abnormalities (e.g., eczema)

The presence of more than five of these classical clinical features, supplemented by clinical findings of heart murmurs on auscultation and developmental delay, can suggest possible candidates for further genetic analysis and a clinical diagnosis of CFC syndrome with 12q interstitial deletion.

## Conclusions

From the case we have discussed above and the extensive literature review, we would like to conclude that the 12q21 deletion could be a possible syndromic manifestation called the CFC syndrome. The most common features are cardiac anomaly, characteristic facial features (such as a prominent forehead and flattened occiput, sparse hair, retrognathia, bulbous nasal tip, triangular face, low-set ears, and hypertelorism), and cutaneous abnormalities (e.g., eczema).

## References

[1] Rauen KA, Cotter PD, Bitts SM, Cox VA, Golabi M (2000). Cardio-facio-cutaneous syndrome phenotype in an individual with an interstitial deletion of 12q: identification of a candidate region for CFC syndrome. Am J Med Genet.

[2] Rauen KA (2007). Cardiofaciocutaneous Syndrome. https://www.ncbi.nlm.nih.gov/books/NBK1186/.

[3] Meinecke P, Meinecke R (1987). Multiple malformation syndrome including cleft lip and palate and cardiac abnormalities due to an interstitial deletion of chromosome 12q. J Med Genet.

[4] Watson MS, McAllister-Barton L, Mahoney MJ, Breg WR (1989). Deletion (12)(q15q21.2). J Med Genet.

[5] Nyström AM, Ekvall S, Berglund E (2008). Noonan and cardio-facio-cutaneous syndromes: two clinically and genetically overlapping disorders. J Med Genet.

[6] Klein OD, Cotter PD, Schmidt AM (2005). Interstitial deletion of chromosome 12q: genotype-phenotype correlation of two patients utilizing array comparative genomic hybridization. Am J Med Genet A.

[7] James PA, Oei P, Ng D, Kannu P, Aftimos S (2005). Another case of interstitial del(12) involving the proposed cardio-facio-cutaneous candidate region. Am J Med Genet A.

[8] Niclass T, Le Guyader G, Beneteau C (2020). 12q21 deletion syndrome: narrowing the critical region down to 1.6 Mb including SYT1 and PPP1R12A. Am J Med Genet A.

[9] Matsumoto A, Mizuno M, Hamada N (2014). LIN7A depletion disrupts cerebral cortex development, contributing to intellectual disability in 12q21-deletion syndrome. PLoS One.

[10] Riggs E, Shakkour Z, Anderson CL, Carney PR (2022). SYT1-associated neurodevelopmental disorder: a narrative review. Children (Basel).

[11] Hughes JJ, Alkhunaizi E, Kruszka P (2020). Loss-of-function variants in PPP1R12A: from isolated sex reversal to holoprosencephaly spectrum and urogenital malformations. Am J Hum Genet.

